# Supplementary material for: A Hybrid Material Combined Copper Oxide with Graphene for an Oxygen Reduction Reaction in an Alkaline Medium
Source: Molecules. 2019 Jan 26;24(3):441. doi: 10.3390/molecules24030441 (PMC6385168; doi:10.3390/molecules24030441)
Supplement: Supplementary file 1 [file molecules-24-00441-s001.pdf]

Supplementary Information

A hybrid material combined copper oxide with graphene for oxygen reduction reaction in alkaline medium

Jiemei Yu[1, 2], Taizhong Huang[1], Zhankun Jiang[1], Min Sun[1*], Chengchun Tang[2*]

1. School of Chemistry and Chemical Engineering, University of Jinan, 336 West Nanxinzhuang Road, Jinan 250022, China

2. School of Materials Science and Engineering, Hebei University of Technology, 8 of First Road of Dingzigu, Hongqiao District, Tianjin 300130, China

1. Experimental Details

1.1 Instrumentation and Measurements

The products were characterized by powder X-ray diffraction (XRD, Cu Ka irradiation; λ = 0.154 nm) with a SIEMENS D5000 X-ray diffractometer. The crystallite sizes of copper oxide can be calculated by the Scherrer's formula:

$$D = \frac{K\lambda}{\beta\cos\theta} \qquad (1)$$

Where D is the average crystalline size, K is the shape factor being 0.890, λ is the wavelength of X-ray being 0.154 nm for Cu Kα radiation, β is the full width at half maximum of the peak, θ is the diffraction angle of the peak.

The morphology of the synthesized samples was tested by scanning electron microscopy (SEM, JEOL JSM-6701F electron microscope operating at 5 KV). Transmission electron microscopy (TEM) images were examined by a Philips Tecnai 20U-TWIN transmission electron microscope with linear resolution of 0.14 nm and dot resolution of 0.19 nm. Raman spectra tests were conducted by a TriVista™ 555CRS Raman spectrometer at 785 nm. X-ray photoelectron spectroscopy (XPS) data was collected by an

[1]E-mail address: chm_sunm@ujn.edu.cn, Tel: +86(0)15053123169

ESCALABMKII X-ray photoelectron spectrometer (VG Scienta, USA) equipped with a monochromatic Al Kα X-ray source (1486.6 eV). The pressure in the chamber during the measurements was kept at 1×10^{-7} Pa. The analyzer was operated at a pass energy of 50 eV for high resolution scans and at a pass energy of 100 eV for survey scans. The binding energy of the C 1s peak at 284.6 eV was taken as a reference for the binding energy calibration. A background subtraction and peak fitting were deconvolved using the XPS peak fitting software (XPSPEAK41 by Prof. R. W. M. Kwok).

1.2 Electrode preparation and electrochemical tests

5 mg of the prepared catalyst powder was dispersed in the mixture of 450 µL of deionized water and 50 µL of Nafion (5 wt% solution alcohols, DuPont). The mixture was fully sonicated to form a homogeneous ink. Then 5 µL of the ink was dropped onto a glassy carbon (GC) electrode of 3 mm in diameter and fully dried. Cyclic voltammetry measurements were performed using a CHI 760E electrochemical workstation (CH Instrument, USA) by conventional three-electrode cell. The coated glass carbon (GC) electrode is employed as the working electrode, graphite as the counter-electrode, and a saturated calomel electrode (Hg/Hg_2Cl_2) (SCE) as the reference electrode.

Before the ORR tests, cyclic voltammetry (CV) tests were performed from 0.2 to -0.8 V at 5 mV/s in Ar-saturated electrolyte to clean the electrode surface. 20 cycles were carried out to stabilize the current-potential signal. Thereafter, the electrolyte was saturated with oxygen before the start of every experiment by bubbling O_2 at least 30 min, which was maintained over the electrolyte in order to ensure its continued O_2 saturation during the recording. The working electrode was cycled at least 20 cycles before data were recorded at a scan rate of 5 mV/s from 0.2 to -0.8 V vs. Hg/Hg_2Cl_2 in O_2-saturated 0.1 mol/L KOH electrolytes.

The Tafel tests were also conducted at a sweeping rate of 5 mV/s. Rotating disk electrode (RDE) and rotating ring disk electrode (RRDE) tests were performed using a RRDE-3A electrode at the same

sweeping rate. For RRDE tests, the working electrode was a glassy carbon disk (5.61 mm in diameter) and a platinum ring leading to a collection efficiency of the ring disk electrode. The RRDE tests were performed at 1600 rpm in O_2-saturated solution. The Pt ring electrode was polarized at -0.3 V vs. Hg/Hg_2Cl_2 for oxidizing the hydrogen peroxide ion during oxygen reduction at the modified GC disk electrode. All the experiments were carried out in 0.1 mol/L KOH solution at room temperature.

The Tafel tests were also conducted at a sweeping rate of 5 mV/s. the exchange current density was derived from the mass-transport correction using Eq. (2):

$$E = E_0 + \frac{2.303RT}{an_aF}\log i_0 - \frac{2.303RT}{an_aF}\log i_d \qquad (2)$$

Where *E* represents the tested electrode potential, E_0 is the thermodynamics electrode potential, *F* is the Faraday constant, *R* is the ideal gas constant, *T* is the thermodynamic temperature, i_d is the measured current density, and i_0 is the exchange current density.

Rotating disk electrode (RDE) and rotating ring disk electrode (RRDE) tests were performed using a RRDE-3A electrode at the same sweeping rate. For RRDE tests, the working electrode was a glassy carbon disk (5.61 mm in diameter) and a platinum ring leading to a collection efficiency of the ring disk electrode. The RRDE tests were performed at 1600 rpm in O_2-saturated solution. The Pt ring electrode was polarized at -0.3 V vs. Hg/Hg_2Cl_2 for oxidizing the hydrogen peroxide ion during oxygen reduction at the modified GC disk electrode. All the experiments were carried out in 0.1 M KOH solution at room temperature.

For RDE test ,the transferred electron numbers of oxygen reduction could be calculated according to the following *Koutechy–Levich* equation (Kakaei and Hasanpour, 2014):

$$\frac{1}{I} = \frac{1}{I_k} + \frac{1}{Bw^{0.5}} \qquad (3)$$

Where I_k is the kinetic current and *w* is the angular velocity ($w = 2\pi N$, *N* is the linear rotation speed).

B could be determined from the slope of the *K–L* plots based on the *Koutechy–Levich* equation as follows:

$$B = 0.62nF(D_{O_2})^{2/3}v^{-1/6}C_{O_2} \qquad (4)$$

Where *n* represents the transferred electron number, *F* is the Faraday constant (*F* = 96485 C/mol), D_{O_2} is the diffusion coefficient of O_2 in 0.1 M KOH (1.9×10^{-5} cm^2/s), *v* is the kinetic viscosity (0.01 cm^2/s), and C_{O_2} is the bulk concentration of O_2 (1.2×10^{-6} mol/cm^3).

For RRDE test, the ORR percentage of peroxide species and electron transfer numbers with respect to total ORR products on our samples were calculated from the following equations:

$$\%H_2O_2 = 200\times\frac{I_r/N}{I_d + I_r/N} \qquad (5)$$

$$n = 4\times\frac{I_d}{I_d + I_r/N} \qquad (6)$$

Where I_d is the disk current, I_r is the ring current, and *N* is the current collection efficiency of the Pt ring 0.39.

2. Pourbaix Diagrams of Cu-H_2O

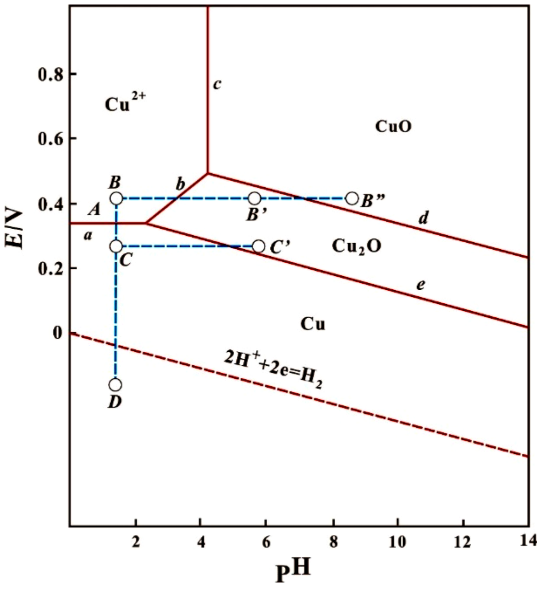

Fig.S1. Pourbaix Diagrams of Cu-H_2O

Reactions on the electrodes:

Cathode: (*C*): $Cu^{2+}+2e^- = Cu$

Anode: (B): $Cu - 2e^- = Cu^{2+}$

a. $Cu^{2+} + 2e^- = Cu$ $E_a = 0.34$ V

b. $2\ Cu^{2+} + H_2O + 2e^- = Cu_2O + 2H^+$ $E_b = 0.20 + 0.059$ pH

c. $Cu^{2+} + H_2O = CuO + 2H^+$ pH = 4.7

d. $CuO + 2H^+ + 2e^- = Cu_2O + H_2O$ $E_d = 0.75 - 0.059$ pH

e. $Cu_2O + 2H^+ + 2e^- = 2Cu + H_2O$ $E_e = 0.47 - 0.059$ pH
